# Kafkaesque systems: An interpretative phenomenological analysis of lived experiences of stateless migrants within the UK

**DOI:** 10.1371/journal.pmen.0000756

**Published:** 2026-10-13

**Authors:** Jessie Mulcaire, Ciarán O’Driscoll, Leah Holt, Sana Zard, Francesca Brady

**Affiliations:** 1 Research Department of Clinical, Educational and Health Psychology, University College London, London, United Kingdom; 2 Helen Bamber Foundation, London, United Kingdom; PLOS: Public Library of Science, UNITED KINGDOM OF GREAT BRITAIN AND NORTHERN IRELAND

## Abstract

Statelessness affects an estimated 4.3 to 10 million people worldwide, denying them access to fundamental rights including education, employment, healthcare, and freedom from arbitrary detention. While stateless individuals are recognised as a marginalised group at elevated risk of human rights violations, discrimination, and trauma, the psychological impact of statelessness remains under-researched. This study aimed to explore how adults with lived experience of statelessness in the UK make sense of its impact on their mental health, and how they access support for any psychological difficulties. A qualitative design using Interpretative Phenomenological Analysis was used. Seven adults affected by statelessness were recruited through non-governmental organisations using purposive sampling. Participants varied in the reasons why they had been affected by statelessness, country of origin, and current immigration status in the UK. Four group experiential themes were generated. Trapped in a Kafkaesque System captured participants’ experience of draconian, discriminatory, and self-perpetuating bureaucratic structures that stripped autonomy, security, and time. Erosion of Self and Belonging described feeling administratively reduced to a non-person, dual legal and relational abandonment, and severed connections. Psychological and Embodied Harm located anxiety, depression, suicidality, post-traumatic stress disorder (PTSD), and somatic symptoms unambiguously within structural conditions, with two participants reporting that UK procedures damaged their mental health more than prior experiences of incarceration; intergenerational effects of the impacts of statelessness were also identified. Survival Strategies and Systemic Limits captured the protective role of community, faith, activism, and equipped services, while emphasising that resilience was bounded and could not substitute for rights. The findings articulate a pathway in which Kafkaesque systems produce entrapment, erode the person’s sense of identity, belonging and connection to others, and generate psychological and embodied harm only partially mediated by coping. Implications include the need for policy reform (reduced waits, restored and adequate Legal Aid funding, the right to work and healthcare access during protracted legal processes), clinician training, and services that recognise statelessness-related distress as legitimate clinical territory rather than deflecting responsibility to immigration authorities.

## Introduction

Stateless people impacted by either what is legally recognised (de jure) and what exists in practice (de facto) are unable to access the privileges, services, protections, and rights that citizens can demand from their governments [1,2]. Current figures range between 4.3 and 10 million residing in 95 countries [3–5] with an increasing number of persons who are stateless or at risk of becoming stateless [6]. Despite campaigns such as ‘#IBelong’, which aimed to eliminate statelessness by 2024, a growing number of states worldwide have introduced legislation allowing for the deprivation of nationality [6–8]. The increasing number of conflicts and the impact of climate change on migration continue to cause disruptions in communities and families, leading to more displacement and more people at risk of statelessness [9,10].

Statelessness happens for many complex reasons. Most people without a nationality belong to marginalized groups facing systemic discrimination; prejudice against race, religion, or ethnic background often shapes exclusionary citizenship laws [7,11,12]. And discrimination within nationality laws can prevent women from passing nationality to their children on the same basis as men, creating a cycle that affects an estimated 70,000 children born each year [5,13–15]. In some cases, statelessness can arise solely from a lack of birth registration; an estimated 230 million children under five have never been registered [12]. Governments may also revoke citizenship to target human rights defenders, journalists, and political opponents [6] and many countries are using national security as a pretext to strip people of their nationality [6].

National and international groups are focusing more on statelessness, as research shows how severely it harms people [16]. Without legal rights, stateless people face even deeper discrimination. They often cannot access education, employment, healthcare, own property, marry, travel or register the birth of a child [11,12,17]. Studies highlight the lack of protection for stateless individuals, leaving them vulnerable to destitution from unemployment without financial assistance [6,16,18].

Stateless people are vulnerable to arbitrary detention for extended periods [5,19]. Notably, the UK is the only country in Europe that permits indefinite immigration detention, with no statutory time limit on the length of time a person may be held. They risk being exploited or trafficked due to legal restrictions and lack of financial aid [17,20–22] particularly during their journey for safety [23]. This creates a cycle: statelessness itself becomes the cause for further stigma and harm [21].

Statelessness is associated with experiences of human rights violations and discrimination and trauma, including exploitation, trafficking, family separation, insecurity, and violence [21,24,25]. These abuses often cause post-traumatic stress disorder and emotional distress [25–27]. We must understand what stateless people endure to help address the mental health problems caused by losing their rights.

The United Nations High Commissioner for Refugees (UNHCR) [3] estimates that 5,483 stateless people live in the UK, though some argue the true total is impossible to know [15,28]. In 2013, the UK created Stateless Determination Procedures (SDP). Under these rules, stateless people can apply for “leave to remain”: permission granted to non-UK nationals to stay lawfully in the UK [17]. While waiting for a decision, applicants should receive help with meeting their basic needs [6]. Yet in the UK, SDP applicants cannot work or receive state-provided financial support and can be at risk of indefinite immigration detention [28,29]. Successful applicants receive a renewable permit to live in the UK and can later apply for citizenship [29]. The SDP is similar to the asylum process, but the burden of proof is higher for statelessness. Charities and international groups have criticized the UK’s system [28,29] because a rejection can leave a person destitute and without rights, unable to travel or apply for status elsewhere [17]; the current UK SDP does not conform to UNHCR recommendations [29] which include allowing appeals, providing legal aid, and independently monitoring Home Office decision-making. Existing research highlights the ways in which these systemic flaws have harmed the mental health of stateless people [5,29].

More broadly, mental health is shaped not only by individual vulnerability but by structural and social determinants, including discrimination, insecurity, and restricted access to material resources [30]. Statelessness, as a status that structurally curtails access to employment, healthcare, and legal protection, can therefore be understood as a social determinant of mental health. Left unresolved, this damages well-being [24,31]. Recent reports link statelessness to depression and suicidal thoughts [5,15] and in some cases, alcoholism [32]. While some UNHCR and non-governmental organisation (NGO) reports mention these psychological effects, researchers have not explored them in detail [5,14]. Existing academic studies suggest the stress of statelessness leads to poorer mental health [31,33]. For example, Rohingya refugees in Bangladesh suffer from PTSD, depression, and physical pain caused by the daily strain of being stateless [25,34]. Comparisons show that citizens are generally happier than stateless people [35]. Herberholz [33] also found some indication that stateless people often have worse mental health than citizens. While we know statelessness harms well-being, we lack deep analysis of its specific psychological effects.

There is also limited available evidence regarding the needs, experiences, and health of stateless people [36] and public awareness is low [37]. While research and policy can highlight personal stories, marginalized groups often struggle to be heard [38]. Researchers argue that research must highlight the voices of people with lived experience to help oppressed groups [39,40].

The UK provides a particularly instructive setting in which to examine the psychological impact of statelessness. It is one of a small number of states operating a formal SDP, offering a defined bureaucratic pathway through which the lived experience of statelessness can be empirically examined. It has also seen an increasingly hostile policy environment toward migration more broadly over the past two decades, raising the question of whether harms attributed to statelessness are distinct from, or compounded by, this wider climate. Access to Legal Aid in the UK has been severely curtailed since the Legal Aid, Sentencing and Punishment of Offenders Act 2012, which removed most immigration matters from scope; subsequent funding cuts have further reduced the availability of free legal representation for individuals pursuing asylum or statelessness claims. Furthermore, much existing evidence on the psychological impact of statelessness derives from Global South and refugee camp contexts (e.g., Rohingya populations in Bangladesh [25,34]); examining statelessness within a high-income, common-law jurisdiction with a distinct legal category for statelessness offers an important and under examined counterpoint.

This study therefore aimed to centre the voices of those with personal experiences of statelessness who were now living in the UK. The aim was to explore how statelessness and legal status affect mental health and secondary to this, any experiences they had of accessing mental health support.

## Materials and methods

### Ethics statement

The study received ethical approval from the University College London (UCL) Research Ethics Committee (22229/001). Informed consent covered audio-recording, anonymised analysis, and the inclusion of verbatim extracts in publications. Participants were assured that participation would have no bearing on the support they received from NGOs or on their legal proceedings. Pseudonyms were used throughout, and any potentially identifying details were altered or removed.

### Design

A qualitative design using Interpretative Phenomenological Analysis (IPA) [41] was employed to explore how adults with lived experience of statelessness in the UK make sense of its impact on their mental health. IPA was selected because of its dual commitment to phenomenology (detailed exploration of lived experience), hermeneutics (the double hermeneutic of the researcher making sense of the Participant making sense of their world), and idiography (close attention to the particular before the general). These commitments align with the study’s aim of generating an experientially grounded, interpretative account of statelessness rather than a descriptive or thematic summary.

### Participants

#### Inclusion and exclusion criteria.

Participants were eligible if they were aged 18 or over and had experience of being considered stateless in the UK, either through legal designation or self-identification. Eligibility was not restricted to those who had pursued the UK SDP, given the small number of individuals to whom this applies. Language was not an exclusion criterion. Individuals were excluded where recruiting NGO staff judged that participation might destabilise their mental health.

#### Sample size and participants.

In keeping with IPA’s idiographic focus, a small, purposive, and reasonably homogenous sample was sought [41]. Sample size was guided by the concept of information power [42], considering the narrow study aim, the specificity of the sample, the quality of dialogue anticipated, and pragmatic factors (time, funding, and the difficulty of building trust with stateless communities). A target of ten participants was set; seven were ultimately interviewed. Nine individuals expressed interest, seven scheduled and attended interviews, and two did not respond to scheduling contacts.

To protect participants, several of whom had ongoing immigration applications, only minimal demographic information was collected, and demographic details are not linked to individual pseudonyms.

Five participants were male, and two were female. Two participants had already received Indefinite Leave to Remain and then British citizenship. One participant held five years’ leave to remain. Four participants were awaiting the outcome of asylum or statelessness determination claims, three of whom had previously had an asylum or statelessness determination claim refused by the Home Office. Ages ranged from 23 to 62 years. One Participant was born in the UK, three in West Asia, one in West Africa, one in central Asia and one in South Asia. Although professional interpreters were available where necessary, all seven participants ultimately conducted their interview in English; for six participants, this was a second language.

### Recruitment

Participants were recruited via NGOs supporting people affected by statelessness, using purposive sampling supplemented by snowballing [41]. NGO staff identified service users who met the inclusion criteria and shared the study information sheet and recruitment poster. Recruitment took place between 18 July 2022 and 01 June 2023. Interested individuals either consented to be contacted by the researcher or contacted the researcher directly.

### Data collection

Information sheets and consent forms detailing the study aims, confidentiality, data protection, and the right to withdraw were provided in advance. Participants could provide audio-recorded verbal consent in lieu of written consent where they had concerns about anonymity or literacy difficulties. Consent was revisited at the start of each interview, and participants were reminded they could withdraw up to the point of analysis.

Each participant took part in one semi-structured interview, conducted between 28 July 2022 and 25 May 2023. Interviews lasted approximately 90 minutes and were conducted online (n = 4), by telephone (n = 2), or in person (n = 1). All were audio-recorded on an encrypted device and transcribed verbatim. The interview schedule was developed collaboratively with an expert by experience (a person with lived experience of statelessness) and clinical psychologists working with stateless people, refugees and other vulnerable migrants. It comprised open, expansive prompts designed to elicit rich, first-person accounts, with follow-up probes used flexibly in line with IPA recommendations.

Participants were debriefed at the end of the interview and given a post-interview information sheet signposting sources of support. Three of the seven participants subsequently reviewed and provided feedback on the overarching group experiential themes as a respondent validation exercise. The three participants who reviewed the group experiential themes confirmed that the themes reflected their experience; their feedback did not lead to the addition or removal of themes but supported minor refinements to the language used, for example strengthening the emphasis on resilience as bounded rather than protective in Theme 4.

### Data analysis

Analysis followed the IPA procedure described by Smith et al. [41], supported by NVivo 12. Each transcript was first analysed individually and in depth, consistent with IPA’s idiographic commitment [41]. This involved repeated close reading of the transcript alongside initial notes and formulating experiential statements capturing the core facets of each participant’s experience through the researcher’s interpretative lens. These statements were then clustered into personal experiential themes for each participant. This process was repeated for each transcript in turn, with the researcher bracketing interpretations from previous cases as far as possible. Once personal experiential themes had been generated for all seven participants, these were compared across cases to identify patterns of convergence and divergence, from which group experiential themes were constructed and refined through iterative re-engagement with the original transcripts.

Quality and validity were addressed with reference to Yardley’s [43] and Nizza et al.’s [44] IPA-specific quality indicators. Reflexive journaling was used throughout, and analytic decisions were discussed in supervision and through cross-coding of extracts with one of the supervising authors.

Sensitivity to context [41,43] was addressed by familiarisation with UK statelessness and immigration law to situate participants’ accounts, and by mitigating power asymmetries through a trauma-informed, compassionate interviewing stance. Additionally, an expert by experience and a panel of stateless individuals consulted on the study’s design and interpretation.

Commitment and rigour were established through prolonged engagement with similar marginalised groups via concurrent NHS clinical work. Data underwent immersive, line-by-line IPA [41], focusing on idiographic depth rather than broad coverage. Interpretations were regularly tested against transcripts and refined through supervisory discussions to prevent premature thematic closure.

Transparency and coherence are demonstrated through detailed procedural reporting and the inclusion of verbatim extracts. Respondent feedback was also sought [45]; three participants reviewed and validated the themes, though the contested nature of member-checking within IPA’s interpretative framework is acknowledged.

### Researcher reflexivity

The first author (JM), who conducted all interviews and led analysis, is a white, working-class Irish woman who migrated to the UK for work prior to training as a clinical psychologist and thus holds the privilege of having migrated legally and of being institutionally located within a discipline shaped by white Western epistemologies.

Bracketing interviews were conducted prior to data collection, which was particularly important given the coincident intensification of UK media and political discourse on migration during the fieldwork period. Reflective supervision supported ongoing examination of her positionality throughout analysis.

Consistent with IPA’s double hermeneutic, the findings represent her interpretation of participants’ meaning-making [41]; the reader, in turn, adds a further hermeneutic layer through their own interpretative engagement with the account [46].

## Results

### Themes

Four group experiential themes were identified that brought together the data from the seven participants and 12 constituent themes. The themes are visually depicted in Fig 1.

**Fig 1:**
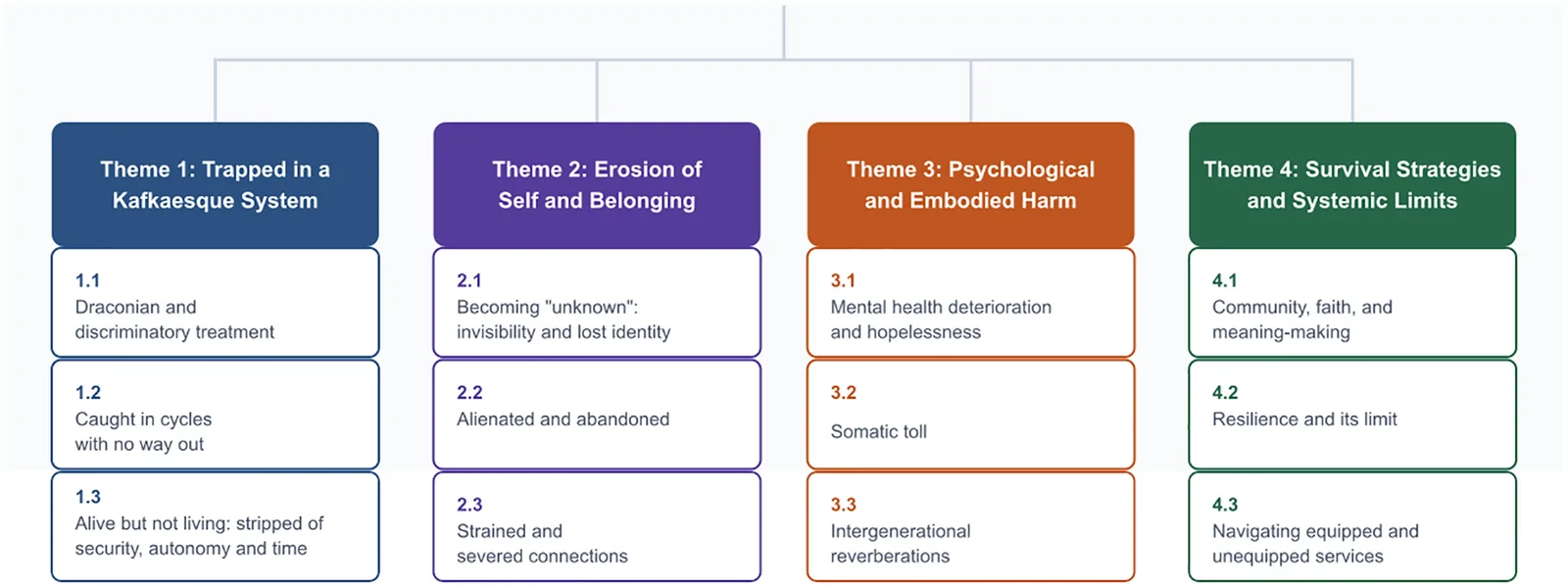
Thematic Map.

### Theme 1: Trapped in a Kafkaesque System

This theme captures participants’ experiences of being caught within bureaucratic structures they described as illogical, hostile, and “nightmarish” (Participant 1). The system was not merely difficult to navigate; it was experienced as actively producing harm through draconian and discriminatory treatment, through cycles that foreclosed any route out, and through the stripping of autonomy, security, and time itself. Participant 1’s choice of the word Kafkaesque anchors the theme: “It’s sort of like there is no way out of this, it’s very Kafkaesque. It’s like I just keep going round and round in circles and that’s the problem with statelessness: having to prove a negative”. Participants described not a system that had failed them, but a system whose internal logic seemed designed to entrap.

#### Draconian and discriminatory treatment.

Participants described the Home Office environment as “more than hostile, draconian” (Participant 1), one in which “everyday people” were licensed to mistreat them. Encounters with caseworkers were experienced as scrutinising and disbelieving: Participant 4 recalled an “angry” official telling them they had “no request to public funds” and should “go back to your home [country].” Participant 6 expressed shock at being told “there is no law for people like you… as long as your life wasn’t in danger… we don’t care,” an exchange that conveyed not only procedural rejection but a denial of moral worth.

For several participants, this systemic hostility intersected with racialised and gendered discrimination. Participant 1 reflected, “if I was white, we would not be having this discussion.” Gender discrimination in nationality laws was identified as a cause of statelessness for some, while others described communities legally excluded from citizenship in their countries of birth. Participant 5 captured the cumulative effect: “they’ve [stateless people] already started off at the very end of the line, right at the back… it [has] felt like a constant battle to quickly go to the start line and try to start with everyone else.” Draconian treatment was not experienced as bureaucratic indifference but as active, personalised injustice leaving participants feeling both criminalised and dehumanised by the very institutions to which they had turned for protection.

#### Caught in cycles with no way out.

The system’s harm was sustained by what participants experienced as deliberately self-perpetuating cycles, in which the resources needed to escape the situation were inaccessible. Participant 6 described preparing to apply for citizenship at 18, only to discover that the documents required were impossible to obtain as a stateless person: “it hit me like a truck, I can’t even get that and so after that it just was even worse.” This foreclosure extended to sources of support that might otherwise have helped resolve participants’ situations: Legal Aid was inaccessible for some, and waiting lists for NGO support were long, leaving participants without recourse either within or outside the formal legal system.

This sense of foreclosure extended beyond legal procedure into existential terrain. Participant 1 described “drowning but not dying you’re just constantly trying to keep your head above the water… suffocating, very much suffocating.” Several participants noted they could neither remain in the UK with rights nor return to their country of birth; even voluntary return was administratively impossible. The cycle threatened to extend across generations: participants with children born in the UK feared their children would inherit the same statelessness, and those with revoked citizenship described an enduring vigilance that their British citizenship might also be removed. Participants did not lack the will to act but acted within a system that they felt had pre-emptively closed every route.

#### Alive but not living: Stripped of security, autonomy and time.

Living within these cycles produced an experience of suspended life. Participant 4 described being in “…limbo. So that impacts a lot you know, so being honest, like saying like it’s a piece of paper, but it’s a life you know. It’s a non-living thing which has a life [documentation]”. Without documentation, participants could not work, study, plan, or travel. Participant 2, who had been imprisoned in their country of birth, reflected: “even when I was in prison, I was much more active.” The legal routes to gaining right to remain in the UK, in their account, was experienced as a more profound erasure of agency than incarceration.

Security and autonomy were experienced as inseparable from citizenship. Participant 5 said: “never, never, never in our entire life for the first decade of my life, I did not have that security.” For those with revoked citizenship, the loss was sudden and incomprehensible, “without knowing the reason behind it for that, it becomes so much worrying at the same time it be so much shock” (Participant 3). For those still in legal procedures, choices narrowed to the point of disappearance: “I don’t have a choice, I can’t go back they don’t have any plans for people like me. Like, what’s the plan? Like you don’t wanna give me the right to stay, you can’t just leave me in the street, but they do” (Participant 6). There was an experience of time as something done to participants, passing without being lived, eroding identity and future alike.

One Participant offered a counter-position, that autonomy could be reclaimed psychologically even where it was materially denied: “you have the power to don’t [not] be submissive to the conditions that [are] impos[ed] [on] you. [You] can change the conditions not let the conditions change you, [you] can be in control of the surroundings, not let the surrounding[s] be controlling you” (Participant 3). This stance is returned to in theme 4 (Survival Strategies and Systemic Limits); here, it stands as the exception.

### Theme 2: Erosion of self and belonging

Theme 1 captured the system that traps stateless individuals; theme 2 explores the impact of this entrapment on the individual. Participants described experiences of a slow dismantling of who they were and where they belonged; being recast as administratively “unknown”. They described being cut off from the protections that nationality is supposed to confer, and pushed to the margins of their families, communities, and the societies in which they lived. Participant 5’s depiction of statelessness as having “no way to belong on this earth” frames the theme: this was not loneliness in the ordinary sense, but a more fundamental ontological exclusion.

#### Becoming “unknown”: Invisibility and lost identity.

Participants described being administratively reduced to a non-person. Participant 3 was officially recorded as “unknown”; Participant 1 also reflected on the experience of being designated in this way:

“Being… mistreated… not allowed to work, not allowed to study, not allowed to do anything because I don’t exist in their eyes, they referred to me as X unknown. That is very hurtful and degrading.” (Participant 1)

This administrative erasure was compounded by professional and societal unfamiliarity with statelessness itself. Participant 3 described how, in the early years of their case, “the issue of stateless was not… being given attention… [it was] unknown… and there was a lot of practical challenges that I used to face.” To be unknown to the system was, in effect, to be unknown to oneself: participants spoke of feeling like a “burden” (Participant 6) and a “failure” (Participant 7), of being “inferior… because of a piece of paper” (Participant 4).

For two participants whose citizenship had been revoked, the loss of identity was even sharper, it was a sudden transition from being a ‘normal’ citizen to being labelled “an enemy of the state” and “a traitor” ” (To preserve anonymity, no Participant number is attributed to this quote). Identity, in these accounts, was no longer an internal process but rather something that the state would give, withhold, or take away. Bureaucratic invisibility turned into a sense of non-being.

#### Alienated and abandoned.

Belonging was experienced not just as social, but as geographical and legal. Participant 5 articulated this with striking clarity:

“What statelessness means is that you have no way to belong on this earth. This world has been divided by borders and marks, and you don’t belong on either side of those marks, of those borders.” (Participant 5)

This non-belonging was reinforced by dispersal and isolation. Participant 6 described being moved to “such a small town… there was no community for… immigrants” and beginning to become “depressed again.” Without a country, embassy, or government to claim them, participants experienced a profound abandonment. Participant 7 captured the sense of being administratively forsaken: “they never really helped me with anything… no accommodation, no work… I never received anything from them.” One Participant described two decades of destitution, food poisoning from having to eat expired food, and homelessness.

For participants whose citizenship had been revoked, abandonment took yet another form: community members stopped communicating with them for fear of reprisals, producing what one Participant called “a social death” *…* (To preserve anonymity, no Participant number is attributed to this quote). Abandonment was simultaneously legal, geographic, and relational, leaving participants with no institution, place, or community that reliably claimed them.

#### Strained and severed connections.

Even where relationships endured, participants described them as strained by the conditions statelessness imposed. Participant 6 reflected on the difficulty of sustaining friendships from a position of forced stasis:

“Even if you find any friends they’re like having their normal life and they’re like they’re working… and you don’t, and it’s not even easy to like make friends, when you’re in this kind of situation.” (Participant 6)

Connection was undermined in several ways. Trust was difficult to extend after experiences of betrayal by state and society. Disclosure carried risk: one Participant avoided telling others about their situation after previous negative reactions. Low mood led to social withdrawal and physical separation from family was enforced by an inability to travel. Within families, silence sometimes settled over traumatic histories. One Participant noted that their relatives would not discuss the experiences statelessness had inflicted on them due to stigma, leaving them feeling disconnected even from those closest to them. Statelessness eroded the conditions of relational reciprocity. Participants were unable to make plans, share futures, or move toward others, and so connection itself became precarious.

### Theme 3: Psychological and embodied harm

Participants located the source of their psychological distress unambiguously in the policies and procedures governing their status, not in personal vulnerability. This theme captures the deterioration of their mental health, the bodily manifestation of that deterioration, and the way statelessness and its psychological consequences reverberated across generations.

#### Mental health deterioration and hopelessness.

Participants described pervasive anxiety, depression, and intrusive rumination tied directly to the conditions of statelessness. Participant 6’s account conveys the texture of this experience:

“Nothing really gets me away from the thoughts [about being stateless] it’s just it’s just temporary, for like a few hours, and again it always comes back to me… I feel old… I feel like my brain isn’t functioning anymore. I don’t know if it’s the effect of antidepressants… I can’t cheer up… my mood is always like I’m down and no matter how much I try to do something it always comes back to me.” (Participant 6)

Anxiety was intensified by governmental immigration policies and surveillance: weekly reporting, electronic tags, and the threat of detention, which Participant 4 described as “every time we go there [to report] every two weeks, [there is] a fear signing in; you have a fear”. Several participants had been prescribed medication to manage their mental health problems. Several had attempted to take their own lives; in every case, this had followed a protracted wait for a legal decision and a corresponding collapse of hope.

Past experiences of trauma compounded current distress. Two participants reported PTSD symptoms “night terrors, night sweats, nightmares” (Participant 4) that had worsened, not eased, since the commencement of processes to regularise their immigration status in the UK. Two who had previously been imprisoned (not in the UK) reported that the asylum system had damaged their mental health more than incarceration had.

Psychological harm was experienced as systemically produced and sustained. Participants were clear that their suffering was a response to conditions, not a property of themselves. As Participant 4 put it, “only one piece of paper can change the whole thing.”

#### Somatic toll.

Psychological harm was experienced in the body. Participants described disturbed sleep, fatigue, headaches, ‘brain fog,’ memory difficulties, and concentration problems. Weight changes were common during asylum and statelessness determination procedures, one person reported binge eating behaviour and another difficulty with adhering to a special diet to manage their long-term health condition. Participant 2’s account captures the embodied intensity of this period:

“I ended up with automatic negative thoughts and anxiety, depression, spells of cry, binge eating. In the middle of the night, I wake up intensely, I feel like night walking, sleep, go, walking, sleeping, sleeping walk and I go to the cake and biscuit in my room, eat and go back to sleep.” (Participant 2)

The body became a site where the bureaucratic violence of statelessness played out. Somatic symptoms were not secondary to psychological distress, but part of it.

#### Intergenerational reverberations.

For participants whose family members were also affected by statelessness, harm extended beyond the individual lifespan. Participant 5 articulated this most directly:

“There’s generational trauma… people aren’t able to enjoy childhood and enjoy that sense of upbringing from both involved parents, even if they are present, they aren’t able to be fully involved [due to their experiences of statelessness].” (Participant 5)

The preoccupation with survival, the fear of losing rights once gained, and the silences around traumatic histories were described as passing from one generation to the next. participants with children spoke of the fear that their children would inherit not only the legal status but the psychological weight of statelessness.

Although this constituent theme was constructed from two accounts, it reframes statelessness as a harm with temporal depth, extending backward into participants’ own childhoods and forward into their children’s futures.

### Theme 4: Survival strategies and systemic limits

Across the interviews, participants described not only what statelessness did to them, but what they did in response. This theme captures the resources: communal, internal, and professional, that participants drew on to survive their situation. It is positioned at the end of the analysis deliberately: coping is real and meaningful, but participants were unambiguous that it could moderate, not undo, the harms described in themes 1–3.

#### Community, faith, and meaning-making.

Communities of various kinds were described as essential. NGOs were the most consistent source of connection, offering not only practical help but a route into peer support, volunteering, classes, and activism. Participant 2 captured the affective quality of this support:

“I’m lucky that I had some friends from my same country that they went through this before me earlier and they felt like kind of responsible for me… they took me to their families, introduced me to their families and like warmth of the family, the warmth of being around with the same people that you belong, and they tried as much as possible to make me feel belong and connected.” (Participant 2)

Faith communities served a similar function. One Participant who did not believe in God continued to attend church because they believed in “good people” (Participant 7). Another described how a priest “really helped me like mentally” (Participant 6) by offering time and care.

Meaning-making, particularly through activism and advocacy, ran through several accounts. Participant 1 described “advocating for the rights of stateless people, refugees, asylum seekers and migrants… on why they [officials] need to look at some of their policies.” For one Participant, activism had been the very ground on which their citizenship had been revoked, and yet they framed this not as loss but as a refusal to abandon their values. Others held onto meaning through faith, “God wants me to [do] another mission” (Participant 2) or through hope for their children’s futures.

Community, faith, and activism worked by re-establishing belonging and purpose directly countering the alienation and identity loss described in theme 2.

#### Resilience and its limits.

Participants spoke of internal resources: coping strategies, perspective, drawing on past adversity. Participant 3 articulated a position of active psychological agency:

“It is realistic that some big psychological impact will be over you, [but] it… should give you a chance to strength, whatever strong part of your personality, to be flourished.” (Participant 3)

Participants reported that therapy, where it was accessible, provided them with concrete tools to aid coping: behavioural activation, routine, grounding techniques. Participant 1 described becoming “creative and unconventional… in order to fill my time and… feel like I’m contributing something.”

Yet resilience had clear limits, and participants were explicit about them. Prolonged statelessness eroded motivation: educational or activity classes attended in hope were abandoned as hope diminished. Strategies that worked early on became unsustainable over years of waiting for the resolution of a legal claim. Participant 4’s account is the clearest statement of this limit:

“When you, for one or two time of a failure, you can absorb. But if the failure start to be continuous, then you start to lose hope… you don’t start to think anything about life. Only one piece of paper can change the whole thing.” (Participant 4)

Resilience could carry participants through episodes of adversity but could not substitute for the material change of documentation, rights that only the state could grant. Framing coping as the solution risks misreading what participants were saying.

#### Navigating equipped and unequipped services.

Participants’ experiences of formal support or healthcare services were sharply polarised. Statutory mental health services were frequently described as ill-equipped to respond to statelessness, sometimes refusing support altogether on the grounds that the cause of distress was “the Home Office” rather than something clinically actionable. Participant 1 captured the absurdity of this:

“What does your nationality have to do with you having a nervous breakdown? OK, you’re having a nervous breakdown because you’re in a situation… but as soon as you mention anything about nationalities, statelessness… we see why you’re [here], there could be nothing wrong with you, you’re here because of the Home Office.” (Participant 1)

Participant 6 similarly described being told they “don’t need to come here anymore” because their issue was legal, and queried why bereavement or financial stress are deemed legitimate grounds for support while statelessness is not?

Equipped services, by contrast, were characterised by demonstrating an understanding of statelessness, non-judgemental engagement, and integration with wider community resources. Participant 7 captured what made the difference:

“Sometimes you just want to someone to listen… because sometimes when you’re telling people about your experience, they think like you are lying… but for me they [NGO] give me a counselling. We had a community and it was helpful. It was like the family I never had.” (Participant 7)

A further barrier was stigma, both societal misunderstanding of statelessness and internalised stigma about mental health. Participant 5 noted that “people just brand it as something entirely different” because the psychological impact of statelessness is not publicly recognised, and that for some stateless individuals “the question isn’t whether there is [support] or not, it’s whether one is willing to acknowledge that they might need to go to these services.”

Where discernible from participants’ accounts, ‘equipped’ services were typically community-based or NGO/charity-led, while ‘unequipped’ services were more often statutory or public health services. This pattern, while not universal across all accounts, may reflect differences in institutional mandate and familiarity with statelessness as a distinct legal and psychosocial category. Equipped services also understood statelessness as a legitimate cause of suffering, and that recognition was itself therapeutic.

## Discussion

This study aimed to explore the lived experience of statelessness in the UK and its impact on mental health. Participants’ accounts describe two interlinked pathways to psychological harm. The first is a direct psychological pathway: entrapment within Kafkaesque systems (Theme 1) eroded participants’ sense of self and belonging (Theme 2), generating psychological and embodied harm (Theme 3). The second is an indirect structural pathway: the material conditions of being stateless specifically within the UK, denial of the right to work, insecure housing, surveillance, and threat of detention, generated additional psychological harm. These depriving conditions compounded the erosion of identity by stripping individuals of core social roles and stability. Theme 4 demonstrates that resilience and coping could moderate, but not undo, these harm. No degree of community support, faith, or internal resilience could substitute for the material change of documentation and rights. This dual-pathway model resonates with comparable arguments made in relation to the UK asylum process more broadly [47] and underscore that the contextual stressors of being stateless in the UK specifically are central to understanding the harms documented here.

### Trapped in a Kafkaesque system

The first theme captures how participants experienced UK statelessness and asylum procedures as illogical, hostile, and inescapable. Participants described draconian treatment, discriminatory policy and practice, perpetuating cycles with no route out, and the stripping of autonomy, security, and time. These accounts align with Kingston’s [12] argument that discrimination is both a cause and consequence of statelessness and resonate with broader literature on perceived discrimination as a predictor of poor mental and physical health [48,49].

participants’ experience of cycles “with no way out” extends existing reports on the inaccessibility of legal aid, prolonged Statelessness Determination Procedures, and the absence of financial support for those undergoing them [5,20,28,29]. Concerns about the inheritability of statelessness echo calls from the European Network on Statelessness [50] and the Institute on Statelessness and Inclusion [6,7] for legal reform to prevent intergenerational transmission. The increased risk of exploitation participants identified mirrors evidence that statelessness creates structural vulnerability to trafficking and modern slavery [17,20–22].

What this study adds is an experiential account of how these systemic features are lived. participants’ language conveys not bureaucratic frustration but a phenomenology of entrapment. The interpretative significance is that harm is not incidental to the system but produced by it.

### Erosion of self and belonging

The second theme captures what entrapment does to identity and connection. Participants described being administratively reduced to a non-person (“X unknown”), legally abandoned by states and communities, and unable to sustain the relational reciprocity on which connection depends.

These findings extend Belton’s [4] work on the relational and identity-based dimensions of statelessness and align with literature on belonging as a determinant of mental health. A lack of belonging has been associated with increased risk of depression [51] and broader physical and mental health difficulties [52], while emotional closeness to a group supports well-being [53]. The role of language in statelessness, particularly the dehumanising potential of labels such as “illegal” or “unknown”, has been noted by Benswait [54] and Brinham [55], and demonstrate how such labels are not merely descriptive but constitutive of experience.

The novel contribution here is the dual finding that legal abandonment (no protecting state, no embassy, no documentation) and relational abandonment (severed family contact, fear of disclosure, community withdrawal) operate together. For participants who had had citizenship revoked, this dual abandonment was particularly acute, captured in the phrase “social death.” Statelessness, on this evidence, is not only a juridical status but an ontological one.

### Psychological and embodied harm

The third theme locates psychological distress unambiguously in the structural conditions of statelessness rather than in individual vulnerability. Participants described anxiety, depression, intrusive rumination, suicidal ideation, PTSD, and a wide range of somatic symptoms: sleep disturbance, fatigue, brain fog, weight change, and memory difficulty, and attributed these directly to UK procedures.

These findings extend Cotterill’s [31] qualitative study, which similarly reported depression and suicidality among stateless individuals in the UK, by demonstrating the embodied dimension of this harm and locating it within a coherent interpretative model of structural causation. They also align with literature on asylum seekers and refugees, in which both pre- and post-migratory stressors contribute to mental health difficulties [56–59]. Notably, every one of McColl et al.’s [60] “7 Ds”: discrimination, detention, dispersal, destitution, denial of right to work, denial of healthcare, and delayed decisions were present in participants’ accounts. This suggests that, while the legal categories differ, the post-migratory stressor profile for stateless individuals in the UK closely mirrors that of asylum seekers [61].

While the post-migratory stressor profile identified here closely mirrors McColl et al.‘s [60] framework, several features of participants’ accounts appear distinctive to statelessness. Participants described the specific evidential burden of having to ‘prove a negative’: establishing the absence of any nationality, rather than substantiating a claim of persecution. Participants also described the structural impossibility of even voluntary return, since without recognised nationality there was no state to return to, foreclosing an exit route available, at least in principle, to most asylum seekers. Participants raised the fear that their UK born children would inherit their statelessness, a distinct intergenerational legal harm not shared by asylum-seeking populations, whose children typically inherit a nationality. These features suggest that while statelessness and asylum share many structural harms, statelessness introduces additional, categorically distinct sources of entrapment.

A striking finding is that two participants who had been imprisoned in their countries of birth described the UK asylum and statelessness system as more damaging to their mental health than that period of incarceration. This is not, on their account, hyperbole; it reflects the experience of indefinite duration, the inability to plan, and the absence of meaningful activity. Existing research consistently links uncertain legal status to poorer mental health [61–64], and Cotterill’s [31] concept of “existential immobility” captures something of this phenomenology. The present study deepens that account by foregrounding the temporal dimension of harm: time itself becomes a site of injury.

The intergenerational constituent theme, although constructed from a smaller subset of accounts, is interpretatively significant. participants described preoccupation with survival, vigilance about losing rights once gained, and silence around traumatic histories as transmitted across generations. This dimension is underdeveloped in the existing statelessness literature and warrants empirical attention.

### Survival strategies and systemic limits

The fourth theme captures how participants coped in response to these harms, and the resources that helped (or hindered) them to do so. Three resources stood out: communities of belonging (NGO, peer, faith, and activist), internal psychological resources (resilience, perspective, meaning-making), and equipped professional services. These findings align with literature on the protective effects of community [65,66], social support [53,67,68], and meaning-making [69] for marginalised populations, and with evidence that lack of social support is itself a health risk [70].

Two findings warrant particular emphasis. First, participants were explicit that resilience is bounded. Coping strategies that worked early in a case became unsustainable across years of waiting, and several participants noted that even acquiring citizenship would not undo the harm of prolonged statelessness. Framing coping or resilience as a sufficient response to statelessness risks obscuring what participants were unambiguous about: only structural change, documentation, and rights can address the underlying cause. This is consistent with critical psychology critiques of resilience discourse as a depoliticising frame [71].

Second, the polarised experience of statutory versus NGO services demonstrates that recognition is itself therapeutic. Equipped services were not necessarily more resourced; they were services that understood statelessness as a legitimate cause of distress. Statutory services that refused support on the grounds that distress was “because of the Home Office” enacted a second form of erasure, an administrative invisibility now repeated within healthcare. This finding extends existing reports of inadequate mental health provision for stateless individuals [36,72] and supports calls for long-term, intensive intervention modelled on work with refugee populations [73].

### Accessing psychological support

Across participants’ accounts, barriers to accessing psychological support grouped into three areas: statutory services turning them away, treating their distress as an immigration status issue rather than a clinical concern; the inaccessibility of Legal Aid and long NGO waiting lists; and stigma, both societal and internalised, around help-seeking. Facilitators of engagement centred on recognition: services characterised as ‘equipped’ offered non-judgemental engagement, an understanding of statelessness as a legitimate source of distress, and integration with community and peer support, “It was like the family I never had”. These findings suggest that what differentiated helpful from unhelpful support was not resource availability but whether statelessness related distress was recognised as legitimate clinical territory.

### Strengths and limitations

To the authors’ knowledge, this is the first published, peer-reviewed qualitative study to explore the mental health implications of statelessness in the UK through an interpretative, experiential lens. Cotterill’s [31] unpublished doctoral thesis similarly reported depression and suicidality among stateless individuals in the UK; the present study extends this work by employing IPA to generate an explicitly interpretative, structural-to-psychological account grounded in participants’ own sense-making. The use of IPA enabled close attention to the meaning participants made of their experiences and produced interpretative insights, particularly around bounded resilience, the embodiment of bureaucratic harm, and recognition as therapeutic. A key strength of this study is that it centres the direct testimony of individuals with lived experience of statelessness, a population that is notoriously difficult to access for research purposes, and whose voices remain rarely represented in academic literature relative to the scale of harm documented in policy and NGO reports.

Several limitations should be noted. While IPA conventionally calls for a reasonably homogeneous sample [41], the seven participants were heterogeneous in nationality of origin, path into statelessness (e.g., due to circumstances of birth versus citizenship revocation), and current legal stage (citizenship acquired, indefinite leave to remain in the UK, or awaiting determination of a claim to regularise their immigration status). However, they were united by a core experience that satisfies IPA’s homogeneity criterion: they have gone through, or are going through, the UK immigration process as a stateless person. This baseline heterogeneity reflects the diverse nature of statelessness itself, meaning variations among participants can be viewed as different angles on the same phenomenon. Thus, divergence may reflect structural differences in legal trajectories rather than simply experiential variation, making any convergence across cases particularly meaningful. IPA was nevertheless selected because it foregrounds participant voice and supports the close interpretation of marginalised experiences [44,74].

The sample size of seven, while appropriate for IPA [41] and reasonable given the difficulty of recruiting from this disenfranchised population, offer in-depth insight into experiences likely to resonate with others navigating similar systems. Recruitment through NGOs introduces a clear sampling bias. One Participant disclosed that they would only have agreed to participate because of trust built with the recruiting organisation. The experiences of stateless individuals who are not connected to NGOs, those most isolated and possibly most harmed, are therefore likely under-represented.

All interviews were conducted in English, which was a second language for six of the seven participants. While no participant requested interpreter support and all demonstrated fluent English throughout, it is possible that some nuance or depth of expression was constrained by conducting the interview in a non-native language; this should be considered when interpreting the findings.

The same researcher conducted all interviews, transcribed, coded, and produced the interpretative account. While supervisory discussion, consultation with an expert by experience, and respondent feedback reduced individual bias, IPA’s double hermeneutic means that the analysis necessarily reflects the researcher’s positionality. Reflexive journaling, bracketing interviews, and reflective practice groups supported, but cannot eliminate, this influence.

To protect participants, several of whom had ongoing immigration applications at the time of interview, only minimal demographic information was collected, and demographic details are not linked to specific quotes in the analysis. This was an ethically necessary decision, but it carries an analytic cost: it limits the reader’s ability to evaluate the relationship between specific accounts and structural positions (e.g., race, gender, country of origin, legal stage) and constrains the discussion’s capacity to develop an intersectional reading. The trade-off between safety and analytic transparency is acknowledged here as an unavoidable feature of conducting research with this population under current immigration conditions.

### Implications

#### Policy.

The findings have clear implications for UK immigration policy that pertains to the protection of stateless people. Most fundamentally, they suggest that the harms documented here are not incidental to current procedures but produced by them, through delay, opacity, restriction of rights, and the conditional conferral of recognition. Drawing directly on participants’ accounts, reforms such as reduced waiting times in statelessness determination procedures, restored access to Legal Aid, and the right to work while awaiting a decision would be expected to prevent the further deterioration of mental health among stateless people living in the UK. Healthcare should be treated as a human right independent of legal status [75,76] and there should be a review of the inheritability of statelessness, in line with European Network on Statelessness [50] and Institute on Statelessness and Inclusion [6,7] recommendations. Reform of the SDP itself is specific to stateless individuals. Other recommendations, overlap with broader immigration, health, and welfare policy affecting asylum seekers and other precarious migrants; we highlight these given the shared determinants of mental health documented in this study. For more comprehensive recommendations on system reform and the associated economic case for change, see [77]

#### Clinical and service.

The findings indicate that statutory mental health services are frequently ill-equipped to respond to statelessness. Training for clinicians on the legal, social, and psychological dimensions of statelessness, modelled on existing recommendations for work with asylum seekers [60], is needed. Holistic provision combining psychological, social, and practical support [78,79], advocacy as part of clinical role [71], and the integration of peer support and community-based approaches into statutory care are all indicated. Most importantly, services need to recognise statelessness-related distress as legitimate clinical territory, not as a problem of legal status to be deflected back to the Home Office.

#### Research.

Blitz et al. [35] identified the psychosocial impact of statelessness as a neglected area; this study addresses part of that gap. Further research should examine the impact of policy change (e.g., reductions in waiting times, access to work) on mental health outcomes. Researchers must investigate the longer-term mental health trajectory of those who acquire citizenship after prolonged statelessness, as participants suggest that the damage often persists even after legal problems end. We need to develop and evaluate interventions adapted to the specific needs of stateless individuals, including peer support and community-building approaches. We should adopt participatory designs that position stateless individuals and community organisations as co-researchers, addressing the trust and access barriers identified here. An examination of intergenerational transmission of statelessness-related harm is a pertinent research question.

It is also worth noting, as a substantive observation arising from the recruitment process itself, that those hardest to reach for research purposes, individuals with no connection to NGOs, are also likely to be those experiencing the most acute systemic harms documented in this study.

## Conclusion

This study offers the first experiential, interpretative account of how statelessness shapes mental health in the UK. participants’ accounts describe a coherent structural-to-psychological pathway: Kafkaesque systems produce experiences of entrapment, which erode self and belonging and generate psychological and embodied harm; community, faith, activism, and equipped professional support partially mediate this impact but cannot substitute for the rights and recognition that only the state can grant. The findings reframe statelessness-related distress as a legitimate clinical and policy concern and locate the responsibility for change at the level of system, not self.
