# Supplementary material for: Kafkaesque systems: An interpretative phenomenological analysis of lived experiences of stateless migrants within the UK
Source: PLOS Ment Health. 2026 Oct 13;3(10):e0000756. doi: 10.1371/journal.pmen.0000756 (PMC13660923; doi:10.1371/journal.pmen.0000756)
Supplement: Supplementary file 1 [file pmen.0000756.s002.docx]

**We are grateful to the reviewers for helping us to strengthen the clarity, conceptual coherence, and rigour of this manuscript, and believe the proposed changes address all comments raised while preserving the central contribution of the study.**

1. Thank you for uploading your study's underlying data set. Unfortunately, the repository you have noted in your Data Availability statement does not qualify as an acceptable data repository according to PLOS's standards.
 
 At this time, please upload the minimal data set necessary to replicate your study's findings to a stable, public repository (such as figshare or Dryad) and provide us with the relevant URLs, DOIs, or accession numbers that may be used to access these data. For a list of recommended repositories and additional information on PLOS standards for data deposition, please see  
<https://journals.plos.org/mentalhealth/s/recommended-repositories>

**We have added a data availability statement:**

***The data for this article cannot be shared in an unrestricted way due to the privacy concerns of the participants included this study. The consent form approved by the University College London Ethics Review Board did not cover the sharing of data beyond the research team (or as aggregated in publications); this decision was based on strong recommendations from community consultants. To request data underlying this manuscript, please contact the corresponding author. You may also contact the University College London Ethics Review Board for any questions or access queries at*** [***ethics@ucl.ac.uk***](mailto:ethics@ucl.ac.uk) ***(ref: 22229/001).***

2. Please ensure that your Ethics Statement is available in its entirety at the beginning of your Methods section, under a subheading 'Ethics Statement'.

**We have now moved this statement to the beginning of the methods section and renamed it.**

If the reviewer comments include a recommendation to cite specific previously published works, please review and evaluate these publications to determine whether they are relevant and should be cited. There is no requirement to cite these works unless the editor has indicated otherwise. 

**Additional Editor Comments (if provided):**

[Note: HTML markup is below. Please do not edit.]

Reviewers' comments:

Reviewer's Responses to Questions

**Comments to the Author**

1. Does this manuscript meet PLOS Mental Health’s [publication criteria](https://eur01.safelinks.protection.outlook.com/?url=https%3A%2F%2Ftrack.editorialmanager.com%2FCL0%2Fhttps%3A%252F%252Fjournals.plos.org%252Fmentalhealth%252Fs%252Fcriteria-for-publication%2F1%2F010f01a04664db27-30908a08-e5c8-4e31-9157-f2fbfa1dba45-000000%2FHn8ZIzhbzNAa71TwRvR_E4aZsmqczqOog5_98Uu7atg%3D258&data=05%7C02%7Cc.odriscoll%40ucl.ac.uk%7Ccff7538ed9424841a6cb08df04b3bc20%7C1faf88fea9984c5b93c9210a11d9a5c2%7C0%7C0%7C639234842145408809%7CUnknown%7CTWFpbGZsb3d8eyJFbXB0eU1hcGkiOnRydWUsIlYiOiIwLjAuMDAwMCIsIlAiOiJXaW4zMiIsIkFOIjoiTWFpbCIsIldUIjoyfQ%3D%3D%7C0%7C%7C%7C&sdata=d0qPY32ecxNVbQRZPJlvcnc7k3%2BV%2BkDy41kUgt%2FSayw%3D&reserved=0)? Is the manuscript technically sound, and do the data support the conclusions? The manuscript must describe methodologically and ethically rigorous research with conclusions that are appropriately drawn based on the data presented.

Reviewer #1: Yes

Reviewer #2: Yes

Reviewer #3: Yes

2. Has the statistical analysis been performed appropriately and rigorously?

Reviewer #1: N/A

Reviewer #2: N/A

Reviewer #3: Yes

3. Have the authors made all data underlying the findings in their manuscript fully available (please refer to the Data Availability Statement at the start of the manuscript PDF file)?

The [PLOS Data policy](https://eur01.safelinks.protection.outlook.com/?url=https%3A%2F%2Ftrack.editorialmanager.com%2FCL0%2Fhttps%3A%252F%252Fjournals.plos.org%252Fmentalhealth%252Fs%252Fdata-availability%2F1%2F010f01a04664db27-30908a08-e5c8-4e31-9157-f2fbfa1dba45-000000%2FR0TwGCoN0pXZA-Q69iKL3sMaF3sMM3rEIYunUiTAADA%3D258&data=05%7C02%7Cc.odriscoll%40ucl.ac.uk%7Ccff7538ed9424841a6cb08df04b3bc20%7C1faf88fea9984c5b93c9210a11d9a5c2%7C0%7C0%7C639234842145440621%7CUnknown%7CTWFpbGZsb3d8eyJFbXB0eU1hcGkiOnRydWUsIlYiOiIwLjAuMDAwMCIsIlAiOiJXaW4zMiIsIkFOIjoiTWFpbCIsIldUIjoyfQ%3D%3D%7C0%7C%7C%7C&sdata=wHgCbJ0cLWuMHsmfP0kfZKalOh4XDIOTA3%2BZRLdW6%2Bk%3D&reserved=0) requires authors to make all data underlying the findings described in their manuscript fully available without restriction, with rare exception. The data should be provided as part of the manuscript or its supporting information, or deposited to a public repository. For example, in addition to summary statistics, the data points behind means, medians and variance measures should be available. If there are restrictions on publicly sharing data—e.g. participant privacy or use of data from a third party—those must be specified.

Reviewer #1: Yes

Reviewer #2: No

Reviewer #3: Yes

4. Is the manuscript presented in an intelligible fashion and written in standard English?

PLOS Mental Health does not copyedit accepted manuscripts, so the language in submitted articles must be clear, correct, and unambiguous. Any typographical or grammatical errors should be corrected at revision, so please note any specific errors here.

Reviewer #1: Yes

Reviewer #2: Yes

Reviewer #3: Yes

5. Review Comments to the Author

Please use the space provided to explain your answers to the questions above. You may also include additional comments for the author, including concerns about dual publication, research ethics, or publication ethics. (Please upload your review as an attachment if it exceeds 20,000 characters)

**Reviewer #1:** Thank you for the opportunity to review this manuscript. I found this to be a thoughtful and well conducted qualitative study addressing an important and underexplored area within mental health research. The manuscript offers valuable insight into the lived experiences of stateless individuals in the UK, and the use of Interpretative Phenomenological Analysis (IPA) is appropriate for addressing the research question. I also appreciated the authors' attention to reflexivity and the way participants' accounts were integrated throughout the analysis. Overall, I believe this study has the potential to make a meaningful contribution to the literature on statelessness and mental health.

However, I have a few comments that I believe would strengthen the manuscript further.
1. Study aims and presentation of the findings
I felt that one area requiring further attention is the alignment between the stated aims and the presentation of the findings. The manuscript states that it aims to explore both the psychological impact of statelessness and participants' experiences of accessing support for mental health difficulties. While the first aim is explored comprehensively throughout the findings, I felt that the second aim receives comparatively less attention. The section describing participants' experiences with services is informative, but further discussion of barriers to accessing support, facilitators of engagement, and participants' perceptions of helpful and unhelpful services would strengthen the paper. Alternatively, the authors may wish to clarify that access to psychological support represents a secondary focus of the study.

**We thank the reviewer for this observation. We agree that the second aim (is addressed less comprehensively than the first. We and added a synthesising paragraph to the Discussion and made it clear in the introduction that it is a secondary focus.**

**Added to Discussion:**

***Accessing psychological support***

***Across participants' accounts, barriers to accessing psychological support grouped into three areas: disbelief and boundary-drawing by statutory services on the grounds that distress stemmed from immigration status rather than a legitimate clinical concern; the practical inaccessibility of Legal Aid and long NGO waiting lists; and stigma, both societal and internalised, around help-seeking. Facilitators of engagement centred on recognition: services characterised as 'equipped' offered non-judgemental engagement, an understanding of statelessness as a legitimate source of distress, and integration with community and peer support, “It was like the family I never had”. These findings suggest that what differentiated helpful from unhelpful support was not resource availability but whether statelessness related distress was recognised as legitimate clinical territory.***

2. Justification for focusing on the UK
The Introduction provides a helpful overview of the UK context and discusses the Statelessness Determination Procedure. However, I think the rationale for focusing specifically on the UK could be developed further. As statelessness is a global issue, it would be useful to explain more explicitly why the UK provides a particularly relevant setting for this study and how this context adds to the existing international literature.

**We agree the rationale for the UK focus needs strengthening. We have expanded the Introduction to explicitly state why the UK is an analytically useful setting:**

**Added to introduction:**

***The UK provides a particularly instructive setting in which to examine the psychological impact of statelessness. It is one of a small number of states operating a formal Statelessness Determination Procedure, offering a defined bureaucratic pathway through which the lived experience of statelessness can be empirically examined. It has also seen an increasingly hostile policy environment toward migration more broadly over the past two decades, raising the question of whether harms attributed to statelessness are distinct from, or compounded by, this wider climate. Access to Legal Aid in the UK has been severely curtailed since the Legal Aid, Sentencing and Punishment of Offenders Act 2012, which removed most immigration matters from scope; subsequent funding cuts have further reduced the availability of free legal representation for individuals pursuing asylum or statelessness claims. Furthermore, much existing evidence on the psychological impact of statelessness derives from Global South and refugee camp contexts (e.g., Rohingya populations in Bangladesh [25,35]); examining statelessness within a high-income, common-law jurisdiction with a distinct legal category for statelessness offers an important and under examined counterpoint.***

***…Notably, the UK is the only country in Europe that permits indefinite immigration detention, with no statutory time limit on the length of time a person may be held…***

3. Proofreading and consistency
The manuscript is generally well written and easy to follow. However, I noticed several minor grammatical, typographical, and formatting inconsistencies that would benefit from careful proofreading before publication.
In addition, there appears to be an inconsistency in the reported timeline. Recruitment is described as taking place between July 2022 and June 2023, whereas the interviews are reported to have been conducted from June 2022 to May 2023. I would encourage the authors to review these dates to ensure they are reported accurately.

**We thank the reviewer for flagging this. We have proofread and corrected any typographical errors.**

**On checking our records, recruitment ran from 18 July 2022 to 1 June 2023, and interviews were conducted from 28 July 2022 to 25 May 2023 (i.e., interviews began after recruitment opened, not before). We have corrected the reported interview date range accordingly in the Methods section (this also addresses Reviewer #2's related Comment 3).**

Overall, I found this to be an interesting and valuable qualitative study with clear relevance to mental health research and practice. The participants' narratives provide important insight into the psychological impact of statelessness, and I believe the manuscript would be further strengthened by addressing the points outlined above, particularly improving the alignment between the study aims and the discussion of access to psychological support.

**Reviewer #2:** The study addresses an under-researched topic with an appropriate method (IPA). It is important to acknowledge that this is a hard-to-reach population. Methodological integrity is evident throughout: reflexivity and bracketing address the researcher's position, respondent validation tests the interpretations, and the analysis is clearly benchmarked against IPA-specific quality criteria. The themes are well-grounded in participant responses, and the chosen extracts support the interpretations offered. The central argument that the system drives the psychological harm, is easy to follow and has clinical value. Alongside these strengths, there are several conceptual and methodological issues that require attention and which I hope will strengthen the manuscript:
1. Distinguishing statelessness-specific harm from asylum-system harm. The sample includes participants at different legal stages (two with citizenship, one with five years' leave, four awaiting asylum or SDP outcomes), and the Discussion notes that all of McColl et al.'s "7 Ds" for asylum seekers were present in participants' accounts. It is not clear which findings are specific to statelessness and which reflect the UK asylum system more broadly. This can be addressed without new data, though: the manuscript already has findings specific to statelessness rather than shared with asylum seekers; for example, having to ‘prove a negative,’ the impossibility of even voluntary return, and the feared inheritance of statelessness by UK-born children. Perhaps a short paragraph in the Discussion could draw these together to state what is distinctive about the stateless experience.

**Added to discussion:**

***While the post-migratory stressor profile identified here closely mirrors McColl et al.'s [59] framework, several features of participants' accounts appear distinctive to statelessness. Participants described the specific evidential burden of having to 'prove a negative': establishing the absence of any nationality, rather than substantiating a claim of persecution. Participants also described the structural impossibility of even voluntary return, since without recognised nationality there was no state to return to, foreclosing an exit route available, at least in principle, to most asylum seekers. Participants raised the fear that their UK born children would inherit their statelessness, a distinct intergenerational legal harm not shared by asylum-seeking populations, whose children typically inherit a nationality. These features suggest that while statelessness and asylum share many structural harms, statelessness introduces additional, categorically distinct sources of entrapment.***

2. Sample homogeneity. The limitations section addresses the sample's heterogeneity and the note on convergence versus divergence across cases is helpful. One addition may strengthen this further: alongside the rationale of “participant voice” for selecting IPA, the authors may add what unites the sample with respect to the homogeneity principle. It seems to me that all seven participants share the experience of facing the UK immigration system with no nationality; they simply describe it from different stages; some from within the wait, others looking back after gaining status.

**We have added to the Strengths and limitations sections clarifying the basis of homogeneity.**

**Revised:**

***While IPA conventionally calls for a reasonably homogeneous sample [42], the seven participants were heterogeneous in nationality of origin, path into statelessness (e.g. due to circumstances of birth versus citizenship revocation), and current legal stage (citizenship acquired, indefinite leave to remain in the UK, or awaiting determination of a claim to regularise their immigration status). However, they were united by a core experience that satisfies IPA's homogeneity criterion: they have gone through, or are going through, the UK immigration process as a stateless person. This baseline heterogeneity reflects the diverse nature of statelessness itself, meaning variations among participants can be viewed as different angles on the same phenomenon. Thus, divergence may reflect structural differences in legal trajectories rather than simply experiential variation, making any convergence across cases particularly meaningful. IPA was nevertheless selected because it foregrounds participant voice and supports the close interpretation of marginalised experiences [45,73].***

3. Recruitment dates: Recruitment is reported as running "between 18 July 2022 and 01 June 2023," yet interviews were "conducted between June 2022 and May 2023." As written, interviewing began before recruitment.

**As noted above in response to Reviewer #1, this has been checked against source records and corrected. Recruitment: 18 July 2022–1 June 2023; interviews: 28 July 2022–25 May 2023.**

4. Respondent validation. The manuscript reports that three participants reviewed and validated the group experiential themes. It would be helpful to know what this exercise contributed: whether the feedback led to any refinement of the themes or confirmed them as presented. A sentence reporting this would allow readers to see the role the validation played in the analysis.

**We have added the following to Methods, Data Collection:**

***The three participants who reviewed the group experiential themes confirmed that the themes reflected their experience; their feedback did not lead to the addition or removal of themes but supported minor refinements to the language used, for example strengthening the emphasis on resilience as bounded rather than protective in Theme 4.***

5. IPA terminology: The paper uses two different sets of IPA terms. The Methods use the newer terms from Smith & Fieldsend (2021): "experiential statements," "personal experiential themes," "group experiential themes" but the Abstract and Results switch back to the older ones, "superordinate themes" and "sub-themes.". One framework used consistently may be clear for readers.

We have used Smith & Fieldsend (2021) terminology throughout.

**Abstract:** **Revised:** Four group experiential themes were generated.

**Results:** **Revised:** "Four group experiential themes were identified... comprising 12 constituent themes."

6. The "first study" claim needs attention. The Discussion says this is "the first qualitative study to explore the mental health implications of statelessness in the UK through an experiential lens," but the paper also cites Cotterill (2022), a UK qualitative doctoral study reporting depression and suicidality among stateless people. Perhaps, add a note to state what makes this study first (e.g., if Cotterill's is an unpublished thesis and not interpretative in approach).

**We have made the following amendment:**

***To the authors' knowledge, this is the first published, peer-reviewed qualitative study to explore the mental health implications of statelessness in the UK through an interpretative, experiential lens. Cotterill's [32] unpublished doctoral thesis similarly reported depression and suicidality among stateless individuals in the UK; the present study extends this work by employing IPA to generate an explicitly interpretative, structural-to-psychological account grounded in participants' own sense-making.***

7. Authors’ voice. Parts of the paper are written in first-person singular (e.g., the reflexivity statement), but this is a five-author paper and it is unclear which author "I" refers to. A brief note clarifying this would help; "The first author conducted all interviews and analysis; the reflexivity statement is written in her voice"; and "author's knowledge" should become "authors' knowledge."

**We have added a sentence at the start of the Researcher Reflexivity section :**

***The first author (JM), who conducted all interviews and led analysis, is a white, working-class Irish woman who migrated to the UK for work prior to training as a clinical psychologist, and thus holds the privilege of having migrated legally and of being institutionally located within a discipline shaped by white Western epistemologies.***

**We also corrected "author's knowledge" to "authors' knowledge" (Strengths and Limitations section)**

8. A sentence in the Introduction ("does not confirm to UNHCR recommendations [29] recommends allowing appeals…"). Please check

**We thank the reviewer for catching this error.**

**Revised:**

***"the current UK SDP does not conform to UNHCR recommendations [29], which include allowing appeals, providing legal aid, and independently monitoring Home Office decision-making."***

9. Interpreter use. The Methods say interpreters were "provided where necessary," yet all seven participants spoke English; it may help n clarify whether any interview used interpreter support. Clarifying whether any interview used an interpreter, and briefly acknowledging the second-language context may strengthen the transparency of the analysis.

**We have clarified in the Methods that although interpreters were available where necessary, all seven participants ultimately conducted their interview in English**

**Addition (Methods, Participants): *Although professional interpreters were available where necessary, all seven participants ultimately conducted their interview in English; for six participants, this was a second language.***

**Addition (Strengths and Limitations):**

***All interviews were conducted in English, which was a second language for six of the seven participants. While no participant requested interpreter support and all demonstrated fluent English throughout, it is possible that some nuance or depth of expression was constrained by conducting the interview in a non-native language; this should be considered when interpreting the findings.***

**Reviewer #3**: Full comments uploaded as attachment

Overall, this is really important work that shines a light on experiences of being stateless within the UK and the harm it produces for individual mental health and wellbeing. The study presents the results of original results, and the centring of lived experience is a strength that is difficult to do with stateless people.

Whilst the article is interesting, it could be significantly strengthened.

1. The article appears to be based on a thesis, and it would benefit significantly from refining it to read more like a journal article.
   a. Care should be taken with references to the author throughout – in many places the article is written as though it is a single author (eg., “familiarising myself”, “to the author’s knowledge”), yet five authors are listed. Clearly state who did what.

**Addressed as above (Reviewer #2, Comment 7); we have reviewed the full manuscript for consistent attribution.**

b. The section on researcher reflexivity is insightful but could be shortened and integrated elsewhere.

**We have now shortened this section:**

***The first author (JM), who conducted all interviews and led analysis, is a white, working-class Irish woman who migrated to the UK for work prior to training as a clinical psychologist, and thus holds the privilege of having migrated legally and of being institutionally located within a discipline shaped by white Western epistemologies.***

***Bracketing interviews were conducted prior to data collection, which was particularly important given the coincident intensification of UK media and political discourse on migration during the fieldwork period. Reflective supervision supported ongoing examination of her positionality throughout analysis.***

***Consistent with IPA's double hermeneutic, the findings represent her interpretation of participants' meaning making [42]; the reader, in turn, adds a further hermeneutic layer through their own interpretative engagement with the account [47].***

c. The text reads quite formulaic and perhaps could be better integrated throughout to improve the flow, eg., the short interpretative sentences at the end of each sub-theme might be better integrated so that the paragraphs flow better in the results section.

**We have revised the Results section to improve narrative flow while retaining the interpretative content.**

2. The pathway from being stateless in the UK to poor mental health could be better developed – how do the four themes fit together and feed into one another? I understand that there is a direct psychological pathway from ‘being stateless’ via erosion of self and belonging, to poor mental health, but there is also an indirect pathway which is fundamentally about being stateless ‘in the uk’ and the contextual stressors – ie., a pathway via being trapped in a system of threat, discrimination, and material and psychosocial conditions that harm mental health – no right to work, poor housing, etc. Then theme four represents how people cope and resist, but the point it that no amount of resilience can prevent the negative mental health consequences in the face of this system.
   a. Even if you focus on the first psychological pathway of ‘being stateless’, the stressors of being ‘in the uk’ are so severe that they have an impact on mental health which must also be acknowledged.
   b. Clearly state the argument you are making in the introduction.
   c. This paper makes a similar argument of how the UK asylum process harms mental health through psychological, material and political pathways <https://www.sciencedirect.com/science/article/pii/S2666560325000726>

**We thank the reviewer for this observation. We have updated the opening paragraph of the Discussion to outline the pathways.**

***This study aimed to explore the lived experience of statelessness in the UK and its impact on mental health. Participants' accounts describe two interlinked pathways to psychological harm. The first is a direct psychological pathway: entrapment within Kafkaesque systems (Theme 1) eroded participants' sense of self and belonging (Theme 2), generating psychological and embodied harm (Theme 3). The second is an indirect structural pathway: the material conditions of being stateless specifically within the UK, denial of the right to work, insecure housing, surveillance, and threat of detention, generated psychological harm largely independently of any erosion of identity. Theme 4 demonstrates that resilience and coping could moderate, but not undo, these harm. No degree of community support, faith, or internal resilience could substitute for the material change of documentation and rights. This dual-pathway model resonates with comparable arguments made in relation to the UK asylum process more broadly [***[***10.1016/j.ssmmh.2025.100460***](https://doi.org/10.1016/j.ssmmh.2025.100460)***], and underscore that the contextual stressors of being stateless in the UK specifically are central to understanding the harms documented here.***

3. There is good background literature provided, but this could be strengthened by including more on the specific UK context and determinants of mental health.
   a. More can be said of the UK-specific context. Politically, the UK has been explicitly hostile for decades and resulting structural harm has only worsened in different guises.
   b. If discussing detention, a major issue is that the UK is the only country in Europe where immigration detainees can be held indefinitely.
   c. Acknowledge the key overlaps and differences between stateless people, refugees and asylum seekers in the UK. It is important to keep the focus on stateless people, but the categories are not always mutually exclusive and there are many shared experiences and determinants of mental health (eg., removed rights, limbo, living conditions) that are discussed.
   d. There is criticism that the burden of proof is applied unfairly and harsher than appears on paper. This could add more nuance. And this is where experience can shine a light on disparities between policy and reality.
   e. Suggest bringing in some literature on structural determinants of mental health (perhaps in paragraph 3). This is currently missing.
   f. Legal aid is discussed but there is no explanation of this in the background. It is unsurprising that legal aid is inaccessible given how severely and progressively it has been cut.

***We thank the reviewer for these suggestions. In line with Reviewer 1 we haved added a section on the UK context.***

**Additions (Introduction):**

***The UK provides a particularly instructive setting in which to examine the psychological impact of statelessness. It is one of a small number of states operating a formal Statelessness Determination Procedure, offering a defined bureaucratic pathway through which the lived experience of statelessness can be empirically examined. It has also seen an increasingly hostile policy environment toward migration more broadly over the past two decades, raising the question of whether harms attributed to statelessness are distinct from, or compounded by, this wider climate. Access to Legal Aid in the UK has been severely curtailed since the Legal Aid, Sentencing and Punishment of Offenders Act 2012, which removed most immigration matters from scope; subsequent funding cuts have further reduced the availability of free legal representation for individuals pursuing asylum or statelessness claims. Furthermore, much existing evidence on the psychological impact of statelessness derives from Global South and refugee camp contexts (e.g., Rohingya populations in Bangladesh [25,35]); examining statelessness within a high-income, common-law jurisdiction with a distinct legal category for statelessness offers an important and under examined counterpoint.***

***…Notably, the UK is the only country in Europe that permits indefinite immigration detention, with no statutory time limit on the length of time a person may be held…***

***More broadly, mental health is shaped not only by individual vulnerability but by structural and social determinants, including discrimination, insecurity, and restricted access to material resources [30]. Statelessness, as a status that structurally curtails access to employment, healthcare, and legal protection, can therefore be understood as a social determinant of mental health. Left unresolved, this damages well-being [24,32].***

4. In the methods section, the section on data analysis is slightly difficult to follow and would benefit from cutting down the section and using more concise language. It should read so that someone less familiar with IPA can understand.

**We have attempted to reduce the length of this section while retaining methodological detail for transparency.**

**Revised:**

***Analysis followed the IPA procedure described by Smith et al. [42], supported by NVivo 12. Each transcript was first analysed individually and in depth, consistent with IPA's idiographic commitment [42]. This involved repeated close reading of the transcript alongside initial notes and formulating experiential statements capturing the core facets of each participant's experience through the researcher's interpretative lens. These statements were then clustered into personal experiential themes for each participant. This process was repeated for each transcript in turn, with the researcher bracketing interpretations from previous cases as far as possible. Once personal experiential themes had been generated for all seven participants, these were compared across cases to identify patterns of convergence and divergence, from which group experiential themes were constructed and refined through iterative re-engagement with the original transcripts.***

5. Is there any information about why participants are stateless that can be explained in the methods (in the results you mention two people had citizenship revoked and gender discrimination in another place)? Is it possible to provide more narrative descriptions about individuals in the methods (even if disconnected from quotes/pseudonyms)? Eg., an older male who became stateless after having his citizenship revoked; a woman awaiting the outcome on an asylum claim after one previously unsuccessful claim. It would help to bring people’s experiences to life. Although I understand this may not be possible and it is more important to prioritise participants’ safety.

**We reviewed our data to ensure any such additions did not risk participant identifiability, in consultation with the research team, we have not added additional information, only including a level of detail where it does not compromise anonymity, consistent with our ethical commitments.**

6. Methods – “having previously declined asylum or stateless determination claims” – make sure it reads as though it is the system that has refused people, not the individuals declining anything.

**We agree the current phrasing inappropriately implies agency/choice on the part of participants**

**Revised:**

***Four participants were awaiting the outcome of asylum or statelessness determination claims, three of whom had previously had an asylum or statelessness determination claim refused by the Home Office.***

7. In paragraph 1 under theme 1.2., “Legal Aid was inaccessible for some and waiting lists for NGO support were long”– explain how it connects to the rest of the paragraph.

**Revised:** ***This foreclosure extended to sources of support that might otherwise have helped resolve participants' situations: Legal Aid was inaccessible for some, and waiting lists for NGO support were long, leaving participants without recourse either within or outside the formal legal system.***

8. Were ‘equipped’ services more community-led/charity based and ‘non equipped’ more public services? This could be useful to highlight if so.

**Addition (constituent** **theme 4.3):**

***Where discernible from participants' accounts, 'equipped' services were typically community-based or NGO/charity-led, while 'unequipped' services were more often statutory or public health services; this pattern, while not universal across all accounts, may reflect differences in institutional mandate and familiarity with statelessness as a distinct legal and psychosocial category.***

9. No need to capitalise Participants throughout.

**This has been addressed throughout.**

10. Strengths and limitations:
    a. “The use of IPA enabled close attention to the meaning Participants made of their experiences and produced interpretative insights, particularly around 19 bounded resilience, the embodiment of bureaucratic harm, and recognition as therapeutic, that would have been difficult to surface through descriptive or thematic methods.” I would lose the last clause because it is possible to understand individual meaning making through thematic methods.

**Revised:** ***The use of IPA enabled close attention to the meaning participants made of their experiences and produced interpretative insights, particularly around bounded resilience, the embodiment of bureaucratic harm, and recognition as therapeutic.***

b. You do not highlight the clear strength of conducting research with lived experience of statelessness, which is very difficult to do.

**Added:** ***A key strength of this study is that it centres the direct testimony of individuals with lived experience of statelessness, a population that is notoriously difficult to access for research purposes, and whose voices remain rarely represented in academic literature relative to the scale of harm documented in policy and NGO reports.***

c. Rephrase ‘limits transferability to some degree’. The results are not generalisable, but they teach us something important.

**Revised:** ***... offer in-depth insight into experiences likely to resonate with others navigating similar systems.***

d. ‘The people hardest to reach…’ this is important but should probably come in the discussion. The framing reads as though you are introducing a new substantive finding in the strengths and limitations.

**Addition (Discussion: Research):**

***It is also worth noting, as a substantive observation arising from the recruitment process itself, that those hardest to reach for research purposes, individuals with no connection to NGOs, are also likely to be those experiencing the most acute systemic harms documented in this study.***

11. Policy Recommendations
    a. Is there a clear immigration policy reform specific to stateless people over and above for refugees and asylum seekers? Otherwise, the reforms touch on health, welfare, and human rights as well as immigration policy.

**Addition:**

***Reform of the SDP itself is specific to stateless individuals. Other recommendations, overlap with broader immigration, health, and welfare policy affecting asylum seekers and other precarious migrants [76]; we highlight these given the shared determinants of mental health documented in this study.***

b. Suggest reframing ‘recommendations’ slightly… ‘reforms such as X,Y,Z, would prevent the deterioration of mental health amongst stateless people living in the UK.’ Reads as more grounded in your work and less of a jump to broad recommendations.

**Revised: *Drawing directly on participants' accounts, reforms such as reduced waiting times in statelessness determination procedures, restored access to Legal Aid, and the right to work while awaiting a decision would be expected to prevent the further deterioration of mental health among stateless people living in the UK.***

c. Refer to <https://refugeeintegrationuk.com/commission-report/> for comprehensive recommendations for system reform and economic argument.

**Addition: *For more comprehensive recommendations on system reform and the associated economic case for change, see [Refugee Integration UK Commission report].***

Figure Resubmissions:

**We uploaded our figure to PLOS’s NAAS tool and have included the NAAS processed figure**
