# Supplementary material for: Kafkaesque systems: An interpretative phenomenological analysis of lived experiences of stateless migrants within the UK
Source: PLOS Ment Health. 2026 Oct 13;3(10):e0000756. doi: 10.1371/journal.pmen.0000756 (PMC13660923; doi:10.1371/journal.pmen.0000756)
Supplement: Supplementary file 2 [file pmen.0000756.s003.docx]

**We thank the reviewers for their comments. We have made amendments in line with the minor comments.**

**Reviewer #1:** Thank you to the authors for carefully addressing my previous comments. I am satisfied with the revisions made, particularly the clearer positioning of access to mental health support as a secondary focus and the strengthened rationale for focusing specifically on the UK. These revisions have improved the clarity and overall coherence of the manuscript.

I have only one minor point remaining. In the response to reviewers, the authors state that the interview dates were checked against the source records and corrected to 28 July 2022–25 May 2023. However, the revised manuscript still reports that interviews were conducted between 28 June 2022 and 25 May 2023, while recruitment began on 18 July 2022. I believe this may simply be an oversight when transferring the correction to the revised manuscript, and I would ask the authors to amend the interview start date accordingly.

**We have corrected this.**

I also noticed a small number of remaining inconsistencies in the capitalisation of “participant/Participant”. A final proofread would therefore be helpful.

**We have corrected these throughout.**

Otherwise, I am satisfied that my previous comments have been adequately addressed.

**Reviewer #2:** The revisions are satisfactory. All substantive comments have been addressed well. Only minor corrections remain-

Interview dates: The Methods still read "28 June 2022," so interviews appear to begin before recruitment opened (18 July 2022). Acc. to the the response letter, this should read "28 July 2022."

**We have corrected this.**

UNHCR sentence: The Introduction still reads "does not confirm to UNHCR recommendations"; please correct to "conform."

**We have corrected this.**

**Reviewer #3:** Thank you for your time editing this article, it has been strengthened significantly. It flows much better, is enjoyable to read, and is an important contribution to the literature. I particularly like the last line of the conclusion.

I would suggest a few very minor edits that can be addressed quickly:

**1. Introduction, paragraph 6 – briefly add what leave to remain means.**

**We have added this explanation: Under these rules, stateless people can apply for "leave to remain": permission granted to non-UK nationals to stay lawfully in the UK [17].**

2. Discussion, paragraph 1 – The second indirect pathway “generated psychological harm largely independently of any erosion of identity”. Is it not ‘additional’ rather than ‘independent’? … it produces additional harm through depriving social/material conditions conducive to good health… which further compounds the erosion of identity? (things like not having a job/career or a home also erode self-identity)

**We agree that it actively compounds the loss of self-identity by stripping away core social roles. We have revised this :**

**The second is an indirect structural pathway: the material conditions of being stateless specifically within the UK, denial of the right to work, insecure housing, surveillance, and threat of detention, generated additional psychological harm. These depriving conditions compounded the erosion of identity by stripping individuals of core social roles and stability.**

3. Discussion, paragraph 16 – “disbelief and boundary-drawing by statutory….” Very long phrase, can this barrier be expressed more succinctly?

**We have rewritten this phrase:**

**Across participants' accounts, barriers to accessing psychological support grouped into three areas: statutory services turning them away, treating their distress as an immigration status issue rather than a clinical concern; the inaccessibility of Legal Aid and long NGO waiting lists; and stigma, both societal and internalised, around help-seeking**

Many sentences require capitalisation at the start:
- Abstract, second paragraph
- Introduction, paragraph 6
- Ethics statement, line 3
- Data collection, line 2
- Results, theme 1, theme 3, 3.1, 3.3, 4.2
- Discussion, paragraph 3, 4, 5, 12
- Conclusion

**We have corrected these throughout.**
