# Supplementary material for: Kafkaesque systems: An interpretative phenomenological analysis of lived experiences of stateless migrants within the UK
Source: PLOS Ment Health. 2026 Oct 13;3(10):e0000756. doi: 10.1371/journal.pmen.0000756 (PMC13660923; doi:10.1371/journal.pmen.0000756)
Supplement: Peer review history

# Peer review: Decision Letter 0

**DOI:** 10.1371/journal.pmen.0000756.r001

**Authors:** Karli Montague-Cardoso

27 Aug 2026

PMEN-D-26-00304

Kafkaesque systems: An interpretative phenomenological analysis of lived experiences of stateless migrants within the UK

PLOS Mental Health

Dear Dr. O'Driscoll,

Thank you for submitting your manuscript to PLOS Mental Health. I am sorry for the delay. After careful consideration of the reviewer reports, we feel that your paper has merit but does not fully meet PLOS Mental Health’s publication criteria as it currently stands. Therefore, we invite you to submit a revised version of the manuscript that addresses the points raised during the review process.

Please address all of the comments that the reviewers have raised, which you can find at the end of this email and in the attachments.

Please submit your revised manuscript by Oct 26 2026 11:59PM. If you will need more time than this to complete your revisions, please reply to this message or contact the journal office at mentalhealth@plos.org. When you're ready to submit your revision, log on to https://www.editorialmanager.com/pmen/ and select the 'Submissions Needing Revision' folder to locate your manuscript file.

Please include the following items when submitting your revised manuscript:

- A letter that responds to each point raised by the editor and reviewer(s). You should upload this letter as a separate file labeled 'Response to Reviewers'.
- A marked-up copy of your manuscript that highlights changes made to the original version. You should upload this as a separate file labeled 'Revised Manuscript with Track Changes'.
- An unmarked version of your revised paper without tracked changes. You should upload this as a separate file labeled 'Manuscript'.

Guidelines for resubmitting your figure files are available below the reviewer comments at the end of this letter.

As the corresponding author, your ORCID iD is verified in the submission system and will appear in the published article. PLOS supports the use of ORCID, and we encourage all coauthors to register for an ORCID iD and use it as well. Please encourage your coauthors to verify their ORCID iD within the submission system before final acceptance, as unverified ORCID iDs will not appear in the published article. *Only*  the individual author can complete the verification step; PLOS staff *cannot*  verify ORCID iDs on behalf of authors.

We look forward to receiving your revised manuscript.

Kind regards,

Karli Montague-Cardoso

Staff Editor

PLOS Mental Health

**Journal Requirements:**

1. Thank you for uploading your study's underlying data set. Unfortunately, the repository you have noted in your Data Availability statement does not qualify as an acceptable data repository according to PLOS's standards.

At this time, please upload the minimal data set necessary to replicate your study's findings to a stable, public repository (such as figshare or Dryad) and provide us with the relevant URLs, DOIs, or accession numbers that may be used to access these data. For a list of recommended repositories and additional information on PLOS standards for data deposition, please see

https://journals.plos.org/mentalhealth/s/recommended-repositories

2. Please ensure that your Ethics Statement is available in its entirety at the beginning of your Methods section, under a subheading 'Ethics Statement'.

If the reviewer comments include a recommendation to cite specific previously published works, please review and evaluate these publications to determine whether they are relevant and should be cited. There is no requirement to cite these works unless the editor has indicated otherwise.

**Additional Editor Comments (if provided):**

[Note: HTML markup is below. Please do not edit.]

Reviewers' comments:

Reviewer's Responses to Questions

**Comments to the Author**

1. Does this manuscript meet PLOS Mental Health’s publication criteria?

Reviewer #1: Yes

Reviewer #2: Yes

Reviewer #3: Yes

**********

2. Has the statistical analysis been performed appropriately and rigorously?

Reviewer #1: N/A

Reviewer #2: N/A

Reviewer #3: Yes

**********

3. Have the authors made all data underlying the findings in their manuscript fully available (please refer to the Data Availability Statement at the start of the manuscript PDF file)?

The PLOS Data policy

Reviewer #1: Yes

Reviewer #2: No

Reviewer #3: Yes

**********

4. Is the manuscript presented in an intelligible fashion and written in standard English?

Reviewer #1: Yes

Reviewer #2: Yes

Reviewer #3: Yes

**********

**Reviewer #1:**  Thank you for the opportunity to review this manuscript. I found this to be a thoughtful and well conducted qualitative study addressing an important and underexplored area within mental health research. The manuscript offers valuable insight into the lived experiences of stateless individuals in the UK, and the use of Interpretative Phenomenological Analysis (IPA) is appropriate for addressing the research question. I also appreciated the authors' attention to reflexivity and the way participants' accounts were integrated throughout the analysis. Overall, I believe this study has the potential to make a meaningful contribution to the literature on statelessness and mental health.

However, I have a few comments that I believe would strengthen the manuscript further.

1. Study aims and presentation of the findings

I felt that one area requiring further attention is the alignment between the stated aims and the presentation of the findings. The manuscript states that it aims to explore both the psychological impact of statelessness and participants' experiences of accessing support for mental health difficulties. While the first aim is explored comprehensively throughout the findings, I felt that the second aim receives comparatively less attention. The section describing participants' experiences with services is informative, but further discussion of barriers to accessing support, facilitators of engagement, and participants' perceptions of helpful and unhelpful services would strengthen the paper. Alternatively, the authors may wish to clarify that access to psychological support represents a secondary focus of the study.

2. Justification for focusing on the UK

The Introduction provides a helpful overview of the UK context and discusses the Statelessness Determination Procedure. However, I think the rationale for focusing specifically on the UK could be developed further. As statelessness is a global issue, it would be useful to explain more explicitly why the UK provides a particularly relevant setting for this study and how this context adds to the existing international literature.

3. Proofreading and consistency

The manuscript is generally well written and easy to follow. However, I noticed several minor grammatical, typographical, and formatting inconsistencies that would benefit from careful proofreading before publication.

In addition, there appears to be an inconsistency in the reported timeline. Recruitment is described as taking place between July 2022 and June 2023, whereas the interviews are reported to have been conducted from June 2022 to May 2023. I would encourage the authors to review these dates to ensure they are reported accurately.

Overall, I found this to be an interesting and valuable qualitative study with clear relevance to mental health research and practice. The participants' narratives provide important insight into the psychological impact of statelessness, and I believe the manuscript would be further strengthened by addressing the points outlined above, particularly improving the alignment between the study aims and the discussion of access to psychological support.

**Reviewer #2:**  The study addresses an under-researched topic with an appropriate method (IPA). It is important to acknowledge that this is a hard-to-reach population. Methodological integrity is evident throughout: reflexivity and bracketing address the researcher's position, respondent validation tests the interpretations, and the analysis is clearly benchmarked against IPA-specific quality criteria. The themes are well-grounded in participant responses, and the chosen extracts support the interpretations offered. The central argument that the system drives the psychological harm, is easy to follow and has clinical value. Alongside these strengths, there are several conceptual and methodological issues that require attention and which I hope will strengthen the manuscript:

1. Distinguishing statelessness-specific harm from asylum-system harm. The sample includes participants at different legal stages (two with citizenship, one with five years' leave, four awaiting asylum or SDP outcomes), and the Discussion notes that all of McColl et al.'s "7 Ds" for asylum seekers were present in participants' accounts. It is not clear which findings are specific to statelessness and which reflect the UK asylum system more broadly. This can be addressed without new data, though: the manuscript already has findings specific to statelessness rather than shared with asylum seekers; for example, having to ‘prove a negative,’ the impossibility of even voluntary return, and the feared inheritance of statelessness by UK-born children. Perhaps a short paragraph in the Discussion could draw these together to state what is distinctive about the stateless experience.

2. Sample homogeneity. The limitations section addresses the sample's heterogeneity and the note on convergence versus divergence across cases is helpful. One addition may strengthen this further: alongside the rationale of “participant voice” for selecting IPA, the authors may add what unites the sample with respect to the homogeneity principle. It seems to me that all seven participants share the experience of facing the UK immigration system with no nationality; they simply describe it from different stages; some from within the wait, others looking back after gaining status.

3. Recruitment dates: Recruitment is reported as running "between 18 July 2022 and 01 June 2023," yet interviews were "conducted between June 2022 and May 2023." As written, interviewing began before recruitment.

4. Respondent validation. The manuscript reports that three participants reviewed and validated the group experiential themes. It would be helpful to know what this exercise contributed: whether the feedback led to any refinement of the themes or confirmed them as presented. A sentence reporting this would allow readers to see the role the validation played in the analysis.

5. IPA terminology: The paper uses two different sets of IPA terms. The Methods use the newer terms from Smith & Fieldsend (2021): "experiential statements," "personal experiential themes," "group experiential themes" but the Abstract and Results switch back to the older ones, "superordinate themes" and "sub-themes.". One framework used consistently may be clear for readers.

6. The "first study" claim needs attention. The Discussion says this is "the first qualitative study to explore the mental health implications of statelessness in the UK through an experiential lens," but the paper also cites Cotterill (2022), a UK qualitative doctoral study reporting depression and suicidality among stateless people. Perhaps, add a note to state what makes this study first (e.g., if Cotterill's is an unpublished thesis and not interpretative in approach).

7. Authors’ voice. Parts of the paper are written in first-person singular (e.g., the reflexivity statement), but this is a five-author paper and it is unclear which author "I" refers to. A brief note clarifying this would help; "The first author conducted all interviews and analysis; the reflexivity statement is written in her voice"; and "author's knowledge" should become "authors' knowledge."

8. A sentence in the Introduction ("does not confirm to UNHCR recommendations [29] recommends allowing appeals…"). Please check

9. Interpreter use. The Methods say interpreters were "provided where necessary," yet all seven participants spoke English; it may help n clarify whether any interview used interpreter support. Clarifying whether any interview used an interpreter, and briefly acknowledging the second-language context may strengthen the transparency of the analysis.

**Reviewer #3** : Full comments uploaded as attachment

Overall, this is really important work that shines a light on experiences of being stateless within the UK and the harm it produces for individual mental health and wellbeing. The study presents the results of original results, and the centring of lived experience is a strength that is difficult to do with stateless people.

Whilst the article is interesting, it could be significantly strengthened.

**********

what does this mean?). If published, this will include your full peer review and any attached files.

**Do you want your identity to be public for this peer review?**  If you choose “no”, your identity will remain anonymous but your review may still be made public.

For information about this choice, including consent withdrawal, please see our Privacy Policy

Reviewer #1: No

Reviewer #2: No

Reviewer #3: No

**********

[NOTE: If reviewer comments were submitted as an attachment file, they will be attached to this email and accessible via the submission site. Please log into your account, locate the manuscript record, and check for the action link "View Attachments". If this link does not appear, there are no attachment files.]

**Figure Resubmissions:**

# Author comment: Author response to Decision Letter 1

**DOI:** 10.1371/journal.pmen.0000756.r002

2 Sep 2026

Attachment Submitted filename: response to reviewers.docx

# Peer review: Decision Letter 1

**DOI:** 10.1371/journal.pmen.0000756.r003

**Authors:** Karli Montague-Cardoso

24 Sep 2026

PMEN-D-26-00304R1

Kafkaesque systems: An interpretative phenomenological analysis of lived experiences of stateless migrants within the UK

PLOS Mental Health

Dear Dr. O'Driscoll,

Thank you for submitting your revised manuscript to PLOS Mental Health. As you will see, the reviewers found the revised version to be largely improved and this will soon be ready for acceptance, pending some minor revisions in response to the comments below. Given their minor nature, when the revised version is submitted, it will be assessed by our team rather than being sent back to reviewers.

Please submit your revised manuscript by Oct 23 2026 11:59PM. If you will need more time than this to complete your revisions, please reply to this message or contact the journal office at mentalhealth@plos.org. When you're ready to submit your revision, log on to https://www.editorialmanager.com/pmen/ and select the 'Submissions Needing Revision' folder to locate your manuscript file.

Please include the following items when submitting your revised manuscript:

- A letter that responds to each point raised by the editor and reviewer(s). You should upload this letter as a separate file labeled 'Response to Reviewers'.
- A marked-up copy of your manuscript that highlights changes made to the original version. You should upload this as a separate file labeled 'Revised Manuscript with Track Changes'.
- An unmarked version of your revised paper without tracked changes. You should upload this as a separate file labeled 'Manuscript'.

Guidelines for resubmitting your figure files are available below the reviewer comments at the end of this letter.

As the corresponding author, your ORCID iD is verified in the submission system and will appear in the published article. PLOS supports the use of ORCID, and we encourage all coauthors to register for an ORCID iD and use it as well. Please encourage your coauthors to verify their ORCID iD within the submission system before final acceptance, as unverified ORCID iDs will not appear in the published article. *Only*  the individual author can complete the verification step; PLOS staff *cannot*  verify ORCID iDs on behalf of authors.

We look forward to receiving your revised manuscript.

Kind regards,

Karli Montague-Cardoso

Staff Editor

PLOS Mental Health

**Journal Requirements:**

If the reviewer comments include a recommendation to cite specific previously published works, please review and evaluate these publications to determine whether they are relevant and should be cited. There is no requirement to cite these works unless the editor has indicated otherwise.

Please review your reference list to ensure that it is complete and correct. If you have cited papers that have been retracted, please include the rationale for doing so in the manuscript text, or remove these references and replace them with relevant current references. Any changes to the reference list should be mentioned in the rebuttal letter that accompanies your revised manuscript. If you need to cite a retracted article, indicate the article’s retracted status in the References list and also include a citation and full reference for the retraction notice.

[Note: HTML markup is below. Please do not edit.]

Reviewers' comments:

Reviewer's Responses to Questions

**Comments to the Author**

Reviewer #1: All comments have been addressed

Reviewer #2: All comments have been addressed

Reviewer #3: All comments have been addressed

**********

publication criteria?

Reviewer #1: Yes

Reviewer #2: Yes

Reviewer #3: Yes

**********

3. Has the statistical analysis been performed appropriately and rigorously?

Reviewer #1: Yes

Reviewer #2: N/A

Reviewer #3: N/A

**********

4. Have the authors made all data underlying the findings in their manuscript fully available (please refer to the Data Availability Statement at the start of the manuscript PDF file)?

The PLOS Data policy

Reviewer #1: Yes

Reviewer #2: Yes

Reviewer #3: Yes

**********

5. Is the manuscript presented in an intelligible fashion and written in standard English?

Reviewer #1: Yes

Reviewer #2: Yes

Reviewer #3: Yes

**********

**Reviewer #1:**  Thank you to the authors for carefully addressing my previous comments. I am satisfied with the revisions made, particularly the clearer positioning of access to mental health support as a secondary focus and the strengthened rationale for focusing specifically on the UK. These revisions have improved the clarity and overall coherence of the manuscript.

I have only one minor point remaining. In the response to reviewers, the authors state that the interview dates were checked against the source records and corrected to 28 July 2022–25 May 2023. However, the revised manuscript still reports that interviews were conducted between 28 June 2022 and 25 May 2023, while recruitment began on 18 July 2022. I believe this may simply be an oversight when transferring the correction to the revised manuscript, and I would ask the authors to amend the interview start date accordingly.

I also noticed a small number of remaining inconsistencies in the capitalisation of “participant/Participant”. A final proofread would therefore be helpful.

Otherwise, I am satisfied that my previous comments have been adequately addressed.

**Reviewer #2:**  The revisions are satisfactory. All substantive comments have been addressed well. Only minor corrections remain-

Interview dates: The Methods still read "28 June 2022," so interviews appear to begin before recruitment opened (18 July 2022). Acc. to the the response letter, this should read "28 July 2022."

UNHCR sentence: The Introduction still reads "does not confirm to UNHCR recommendations"; please correct to "conform."

**Reviewer #3:**  Thank you for your time editing this article, it has been strengthened significantly. It flows much better, is enjoyable to read, and is an important contribution to the literature. I particularly like the last line of the conclusion.

I would suggest a few very minor edits that can be addressed quickly:

1. Introduction, paragraph 6 – briefly add what leave to remain means.

2. Discussion, paragraph 1 – The second indirect pathway “generated psychological harm largely independently of any erosion of identity”. Is it not ‘additional’ rather than ‘independent’? … it produces additional harm through depriving social/material conditions conducive to good health… which further compounds the erosion of identity? (things like not having a job/career or a home also erode self-identity)

3. Discussion, paragraph 16 – “disbelief and boundary-drawing by statutory….” Very long phrase, can this barrier be expressed more succinctly?

Many sentences require capitalisation at the start:

- Abstract, second paragraph

- Introduction, paragraph 6

- Ethics statement, line 3

- Data collection, line 2

- Results, theme 1, theme 3, 3.1, 3.3, 4.2

- Discussion, paragraph 3, 4, 5, 12

- Conclusion

**********

what does this mean?). If published, this will include your full peer review and any attached files.

**Do you want your identity to be public for this peer review?**  If you choose “no”, your identity will remain anonymous but your review may still be made public.

For information about this choice, including consent withdrawal, please see our Privacy Policy

Reviewer #1: No

Reviewer #2: No

Reviewer #3: No

**********

[NOTE: If reviewer comments were submitted as an attachment file, they will be attached to this email and accessible via the submission site. Please log into your account, locate the manuscript record, and check for the action link "View Attachments". If this link does not appear, there are no attachment files.]

**Figure Resubmissions:**

# Author comment: Author response to Decision Letter 2

**DOI:** 10.1371/journal.pmen.0000756.r004

25 Sep 2026

Attachment Submitted filename: response - minor.docx

# Editor report: Decision Letter 2

**DOI:** 10.1371/journal.pmen.0000756.r005

**Authors:** Karli Montague-Cardoso

29 Sep 2026

Kafkaesque systems: An interpretative phenomenological analysis of lived experiences of stateless migrants within the UK

PMEN-D-26-00304R2

Dear Dr O'Driscoll,

We are pleased to inform you that your manuscript 'Kafkaesque systems: An interpretative phenomenological analysis of lived experiences of stateless migrants within the UK' has been provisionally accepted for publication in PLOS Mental Health.

Before your manuscript can be formally accepted you will need to complete some formatting changes, which you will receive in a follow up email. A member of our team will be in touch with a set of requests.

Please note that your manuscript will not be scheduled for publication until you have made the required changes, so a swift response is appreciated.

IMPORTANT: The editorial review process is now complete. PLOS will only permit corrections to spelling, formatting or significant scientific errors from this point onwards. Requests for major changes, or any which affect the scientific understanding of your work, will cause delays to the publication date of your manuscript.

If your institution or institutions have a press office, please notify them about your upcoming paper to help maximize its impact. If they'll be preparing press materials, please inform our press team as soon as possible -- no later than 48 hours after receiving the formal acceptance. Your manuscript will remain under strict press embargo until 2 pm Eastern Time on the date of publication. For more information, please contact mentalhealth@plos.org.

Thank you again for supporting Open Access publishing; we are looking forward to publishing your work in PLOS Mental Health.

Best regards,

Karli Montague-Cardoso

Staff Editor

PLOS Mental Health

***********************************************************

Reviewer Comments (if any, and for reference):
